# Static Guided Endodontics in Primary Endodontic Treatment of Anterior Teeth: A Narrative Review

**DOI:** 10.3390/dj14040195

**Published:** 2026-03-26

**Authors:** Monika Kuczmaja, Wiesława Puchalska, Agata Żółtowska

**Affiliations:** Department of Conservative Dentistry, Faculty of Medicine, Medical University of Gdańsk, 80-210 Gdańsk, Poland; monika.kuczmaja@gumed.edu.pl (M.K.); w.puchalska@onet.eu (W.P.)

**Keywords:** cone beam computed tomography, endodontics, guided access, guided endodontics, pulp canal obliteration, static navigation

## Abstract

**Background**: Guided endodontics (GE), introduced in 2016, is an innovative approach aimed at addressing the challenges faced in endodontic treatment, particularly in cases of pulp canal obliteration (PCO). **Objectives**: This narrative review aims to assess the efficacy and application of static guided endodontics to facilitate minimally invasive access to difficult-to-locate root canals during primary endodontic treatment of incisors and canines. **Method**: A search strategy of the literature was performed on PubMed until 18 November 2025. The review synthesizes findings from 28 studies, focusing on recent advancements, procedural planning, and clinical outcomes related to GE. **Results**: Key findings indicate that GE may improve the ability to locate and treat calcified canals, reduce complications associated with traditional methods. Radiographic assessments and clinical indicators demonstrate favorable short- to medium-term outcomes; however, there is an absence of standardized protocols for long-term follow-up. **Conclusions**: Recommendations for future research include the establishment of unified technical guidelines to enhance consistency and comparability of results across clinical settings. Overall, guided endodontics represents a promising advancement in improving the success of root canal therapy while preserving natural dentition. The primary goal of this article is to update the literature review on static guided endodontics in anterior teeth during primary endodontics.

## 1. Introduction

Endodontic treatment of teeth with completely or partially obliterated root canals presents significant challenges for clinicians. This is due to the increased risk of perforation, difficulty in locating the canal and a higher rate of treatment failure when conventional techniques are used. Locating calcified or obliterated canals can be very time-consuming and technically demanding. It is often associated with a greater likelihood of perforations and instrument fractures [1,2]. These complications can further complicate the procedure and potentially compromise the structural integrity of the tooth [3]. Despite these difficulties, it remains worthwhile to attempt root canal therapy rather than extracting the tooth. Preserving natural teeth helps maintain function, aesthetics, and overall oral health, and can also prevent additional oral ecosystem complications. The oral cavity is the start of digestion and affects speech, function, health, and appearance, with tooth loss impairing self-esteem and social interactions [4]. Tooth loss due to caries or pulp issues disrupts the oral ecosystem, causing localized problems and issues like TMJ pain [5], headaches, and muscle discomfort [6].

A critical factor contributing to such endodontic treatment challenges is pulp canal obliteration (PCO). Dental pulp is the connective tissue which contains different type of cells—odontoblasts, fibroblasts, mesenchymal stem cells, nerve fibers and vessels [7,8]. The structural relationship between dental pulp and dentin is known as the “dentin—pulp complex” [7]. Dentin synthesis and apposition along dental pulp cavity walls occur during the life of a vital tooth and depend on the activity of specialized cell odontoblasts. The primary dentin is produced during tooth development till the apical foramen is closed [9]. The secondary dentin is produced along all pulp cavity walls throughout life and is a physiological aging process [7,9]. The tertiary dentin is deposited on secondary dentin as a response to pathological stimuli such as caries, traumatic injuries, or orthodontic therapy. That process leads to rapid obliteration of the pulp cavity. The tertiary dentin can be divided into reactionary and reparative, depending on the degree of stimuli [7,9]. The pulp canal obliteration (PCO) is defined as a deposition of hard tissues in the root canal space, most often after dental trauma, which leads to reduction in canal diameter [1,2]. The precise mechanism of PCO is still unclear [1,9]. Accelerated apposition of dentin in PCO is possibly related to revascularization and/or reinnervation of the whole pulp or parts of the pulp after reduced blood flow in pulp vessels due to traumatic dental injury (TDI). Differences in patterns of neural and vessels repair can lead to losing control of sympathetic nerve stimulation over the secretory activity of odontoblasts. That results in rapid deposition of dentin. Inhibitory control of odontoblastic secretion can be reinstituted after pulp revascularization is finished—resulting in partial PCO [9,10]. PCO can occur as a response to different stimuli—for example in teeth after dental trauma [1,9], after orthodontic treatment [11], or in patients on long-term glucocorticosteroids [12]. There are reports suggesting that systemic diseases such as thyroid disorders, hypertension, and diabetes may be linked to pulp canal obliteration (PCO). Hormonal changes and metabolic alterations associated with these conditions can affect dental health, leading to structural changes in teeth and contributing to the occurrence of PCO. Further studies are needed to understand these relationships better and their impact on endodontic treatment [13]. According to Su et al. (2023) [14], isolated dentin defects like Dentin Dysplasia Type I (DD-1) and Dentinogenesis Imperfecta (DI) are correlated with Pulp Canal Obliteration (PCO), with these conditions linked to mutations in the DSPP gene. Moreover, several systemic diseases associated with PCO were identified, including Osteogenesis Imperfecta (OI), Hypophosphatemic Rickets, Ehlers-Danlos Syndrome (EDS), and Schimke Immuno-Osteodysplastic Dysplasia (SIOD), which may be associated with specific changes in tooth structure that lead to pulp canal obliteration [14]. Clinical symptoms of PCO may be crown discoloration (darker or yellowish) and lower or negative response to pulp sensibility tests, although the process of calcification is usually asymptomatic [1,3]. Radiographically, PCO can be partial—when the chamber is not detectable, but part of root canal is visible, or when both chamber and canal are hardly or not detectable [2]. The pulp tests express only conductivity of pulp nerve fibers—not pulp vascular supply. The radiographs reveal the result of osteoclastic/osteoblastic activity in the root or bone, not any other pathological or healing event in process in the pulp [10]. The pulp necrosis in teeth with PCO varies from 1 to 27% but is considered low [1]. Thus, most studies suggest that teeth with PCO should be monitored clinically and radiographically. The root canal treatment should be initiated when periapical disease or clinical symptoms occur. PCO is a challenge during endodontic treatment even for dentists specializing in endodontic treatment [1,3].

In response to the challenges posed by pulp canal obliteration (PCO), guided endodontics (GE) was introduced as a pioneering approach. It was first presented by Krastl et al. in 2016 [15] as a novel treatment strategy specifically designed to address the difficulties associated with treating teeth affected by PCO [15]. The aim of guided endodontics is to achieve minimally invasive access for root canals preparation [15] or endodontic microsurgery [16]. The concept is based on the principle of guided surgery used to reach proper implant placement [15,17,18]. A virtual treatment plan in implantology is now a reality and common procedure. Surgical guides facilitate the insertion of dental implants into an ideal position [19,20]. Today, guided endodontics is used in multiple treatments, such as locating root canals in teeth with pulp canal obliteration [15] and locating canal paths in teeth with previous endodontic failure—for example via falsa, deviation [3], removing glass fiber posts [21], treating teeth with complicated anatomy, e.x. dens invaginatus [22], or microsurgical endodontics [23]. Guided endodontics can be divided into static guided endodontics (use of a template) and dynamic guided endodontics, where markers are positioned in patient’s mouth and a stereo camera is connected to the dynamic navigation system [24,25]. Cone-beam computed tomography (CBCT) is the basis of digital planning in guided endodontics, both static and dynamic [17].

The entire process of static guided endodontics can be divided into two main parts:

(1) A planning and laboratory part—digital planning and laboratory production of endodontics guide
(2) A clinical part—endodontics treatment with the use of endodontic guide [17].

The first step of planning demands a CBCT and patient’s arch digital registration. To visualize calcified root canals, a CBCT with the smallest field of view and high resolution is required. The CBCT scan is stored in a Digital Imaging and Communication (DICOM) format. Digital arch registration can be performed directly by an intraoral scanner or by taking an impression and scanning the dental model in the dental laboratory. The digital file of arch registration is stored in Standard Tesselation Language (STL) format. For the virtual planning, the digital planning software is required. Digital software synchronizes and overlays the DICOM file from CBCT with the STL file of the patient’s arch model. The CBCT image should allow to localize the visible part of the root canal. The virtual image of the drill is positioned so that the tip of the drill reaches the visible part of the root canal. Once the drill and sleeve positions are planned, the virtual template is designed. The guide should cover the labial and palatal surfaces of the adjacent teeth; the high of the sleeve and the diameter of the sleeve hole should be adapted to the drill length and diameter. The STL file of the temple is exported from planning software and processed in the slicer software, which allows to prepare the file for 3D printing. The clinical steps start with checking the endodontics guide fitting and stability. The enamel is removed with the diamond bur. A mark placed through the template can indicate the region of the endodontic access. Then, the specific bur is inserted through the prepared hole and moved slowly in depth with pumping movements. The drill is cleaned regularly of debris and the root canal is irrigated. After each few millimeters gain in the depth, the hand file is used to check if the canal can be negotiated. The procedure is continued till the bur is stopped by the sleeve dimension. If the canal is located, the conventional root canal treatment can be performed [15,17,18,24,25,26].

The entire workflow is presented in Table 1.

## 2. Materials and Methods

A search strategy of the literature was performed on PubMed until 18 November 2025 with the following keywords: “Guided Endodontic” OR “Guided Endodontics” OR “Guided Endodontic Access” OR “Guided Endodontic Treatment”. No year restriction nor language restriction were applied. The abstracts were manually reviewed by two independent researchers and articles with exclusion criteria were screened out. Later, full-text evaluation was performed to complete the list of articles.

Inclusion criteria were: specific keywords, the case reports which involve primary endodontic treatments of incisors and canines, and in vivo studies.

Exclusion criteria were: articles in other languages than English, reviews, articles focusing on premolars and molars, in vitro studies, articles on guided endodontics microsurgery/surgery, articles on dynamic guided endodontics, augmented reality, PriciGuide, case reports describing the use of endodontic navigation for endodontic retreatment, removal of fractured instruments, glass fiber post removal or treatment of dens invaginatus, experts’ opinions/comments/responses to letters, and articles that do not focus solely on guided endodontics (GE).

**Figure 1:**
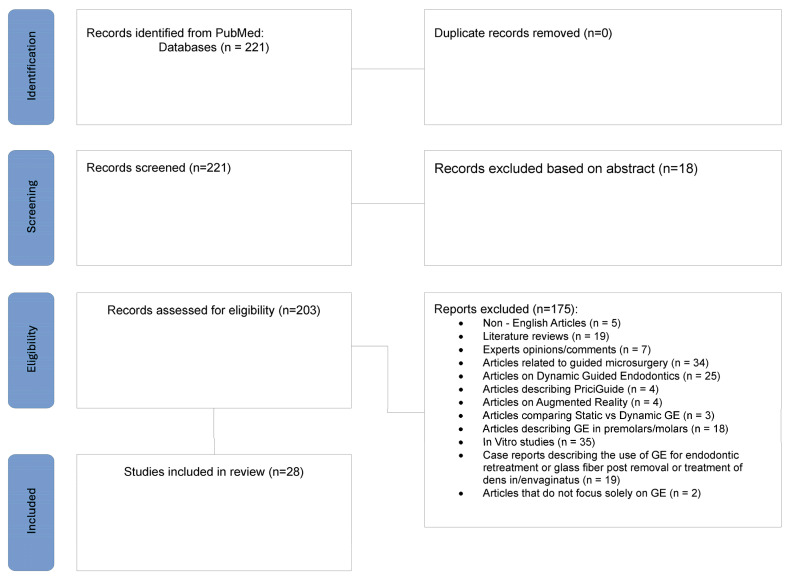
PRISMA flow chart of the selection process.

The PRISMA flow chart of the study selection is presented in Figure 1.

## 3. Results

Based on the literature review, 221 publications were initially identified; 203 articles were selected for full-text review. Subsequently, 175 articles were excluded, leaving a final set of publications for analysis. For the study, case reports and original studies related to guided endodontics (GE) and its applications on anterior teeth, such as incisors and canines, published from 2016 onwards, were focused upon. The selected articles, which address primary indications and techniques of guided endodontic procedures, are summarized in Table 2. These publications encompass a range of approaches and outcomes, providing a comprehensive overview of recent advancements in the field.

Introduction and Objectives of Guided Endodontics Therapy

From the 28 selected studies 27 are case reports/case series studies and one [49] is an observational study in 50 patients. The diagnoses of treated teeth were mainly described as Apical Periodontitis (AAP, SAP, AAA, CAA) or pulp necrosis. All selected studies reported PCO as a main problem and the reason why guided endodontics was applied; additional problems were reported in two studies: root fracture [27] and previous iatrogenic deviation [28].

First Visit and Imaging Diagnostics

The clinical and digital protocols were similar in all studies: the CBCT as the radiographic examination, intraoral scans/plaster casts scanning, and exporting both files to the scanning software and matching them. The CBCT machines used in the studies varied widely. The voxel size is specified in 13 studies and differs from 0.075 mm to 0.5 mm. The CBCT images allowed to visualize part of the root canals, though the planned length of the drill path is not always specified. The conventional intraoral impressions with a following plaster cast scanning were performed in five studies [29,30,31,32,33]; in 23 studies intraoral scans were taken [27,28,34,35,36,37,38,39,40,41,42,43,44,45,46,47,48,49,50,51,52,53].

Procedure Planning and Design

The majority of studies utilized various software solutions for guided endodontic planning, with common tools including BlueSkyPlan [27,35,40,43,48,52], SimPlant [28,33,47,50] and 3Shape [39,46,53]. In addition, endodontic guides were predominantly fabricated using a range of 3D printing technologies, the most commonly used being the Objet Eden 260 V [28,33,50,51] and materials, mainly resins, with some variations in the use of metal or sleeve-less designs.

Clinical Practice: Procedure Execution

A review of current research indicates that various types of drills were employed during clinical procedures for guided endodontics to obtain endodontic access to the patent part of canal. The most commonly used drill was the Munce Discovery Bur (CJM Engineering, Santa Barbara, CA, USA) [27,31,35,36,40,41,52]. Several other drills were utilized: Tivoly drills [34], Gates Glidden drills [42], and EG5 drills [29]. Most studies reported using a drill speed of around 10,000 rpm [31,33,34,38,43,44,47,51], though some employed higher or lower speeds depending on the bur type or conditions, while many did not specify the drill speed at all [27,35,36,37,39,40,41,42,45,46,48,52,53]. The time needed to locate the obliterated canal with GE is reported only in a few studies and varies from approximately 5 to 15 min [29,32,42,48], while many did not specify the exact time.

Follow-up and Outcome Evaluation

Follow-up periods ranged from 24 h [45] to up to 3 years [29]. In some articles the follow-up periods are very short—days or weeks [28,33,38,45]. The short follow-up periods were not enough to evaluate the radiological outcome. A 6-month follow-up period was reported in five studies [30,31,34,36,41], a one-year period was reported in six studies [27,28,35,42,43,50], an 18-month period was reported in four articles [34,40,42,53], and 2-year and 3-year periods were reported in two cases [29,37]. Some studies did not report any follow-up evaluation [32,46,48,49,51,53].

The clinical outcomes connected with pain were mainly described as “asymptomatic” or “no pain” [28,29,31,33,36,39,40,41,42,43,44,50,52]. Most studies reported teeth as asymptomatic, with normal function and radiographic evidence of healing [27,29,30,31,34,35,36,37,39,40,41,43,44,47,50,52], though several lacked detailed descriptions of healing outcomes [28,32,33,38,42,45,46,48,49,51,53].

The clinical and radiological follow-ups are summarized in Table 3.

## 4. Discussion

The main aim of root canal treatment is chemical cleaning and disinfection, which can be performed only when root canals are located, negotiated and mechanically prepared. Missed canals are highly connected with later apical periodontitis [54,55]. The PCO—confirmed in radiograph and CBCT—was the reason for using GE in all reviewed studies. The application of GE in all described cases was described as clinically successful—the obliterated root canals were located, the work length was reached and canals were obturated. The CBCT plays a crucial role in preoperative planning of calcified canals treatment. For the diagnosis of endodontic problems, the small FOV (Field of View) CBCT scans are recommended. In the small FOV scan, the volume of exposed tissues is reduced as well as the scatter, which improves image quality [56]. The recommendation of voxel size in endodontics varies—100 µm or less [56], or 76–300 µm [57]. Only a few studies of the present review reported applied both FOV and voxel size during CBCT [31,32,34,37,38,40,46]. Moreover, studies differ in the reported units of measurement—future directions are required to standardize the CBCT protocol reporting in GE. The use of intraoral scanner reduces the number of steps. However, it is not necessary to achieve good results in guided endodontic planning. A conventional impression and gypsum cast scanning can also be successfully used [29,30,31,32,33]. However, the more steps are taken, the higher amount of errors can occur.

Planning and designing the guided endodontic procedure requires thorough anatomical analysis and careful selection of appropriate instruments, such as burs with suitable diameters and lengths. Not only is space a limitation—consideration of root thickness is also crucial, as it significantly influences instrument choice and access technique. When planning access on mandibular incisors, which have narrower roots compared to maxillary teeth, the use of thinner burs is necessary to prevent structural damage and root fracture, as supported by studies within minimally invasive techniques [58]. In this context, tools such as Munce Discovery Burs (CJM Engineering, Santa Barbara, CA, USA) are highly recommended because they offer high precision and ergonomic design, ensuring better control during drilling and thus reducing the risk of fracture or perforation. These tools are widely endorsed in the literature and used by many researchers in both clinical and experimental studies [59]. When selecting the rotational speed of the instruments, it is essential to control the force applied and prevent excessive heat generation, which can damage periapical tissues and the periodontal ligament. The recommended speed, typically around 10,000 rpm, is supported by scientific evidence indicating that proper parameters enhance both safety and precision in endodontic procedures [15]. Effective cooling during the operation, especially when drilling in calcified canals, is critical to prevent thermal tissue damage and preserve the integrity of adjacent bone and ligament structures [60]. It is also important to highlight that the process of planning and preparation within guided endodontics requires a significant investment of time, although this approach consistently demonstrates a reduction in overall procedure duration—typically from a few to several minutes—which has been confirmed in studies [61,62]. Preparing the template and planning the access usually takes approximately 9.4 min, while performing the actual access can be completed in about 30 s. This not only minimizes tissue damage and reduces patient chair time but also lowers the risk of procedural complications. According to studies by Cvek et al. [63], the total failure rate was 20%, including perforation of the root, fracture of a file, or root canal not found, during root canal treatment on incisors with PCO [63]. During the procedure itself, precise control of drilling speed and cooling, along with the avoidance of excessive pressure and heat build-up, is critical to prevent cavitation, perforations, or damage to soft and hard tissues [1]. The use of advanced technologies such as CAD/CAM systems further enhances accuracy by enabling the production of highly precise, three-dimensional templates, as confirmed by various experimental and clinical studies [64]. Moreover, treating calcified root canals traditionally is highly time-consuming. Guided endodontics allows the procedure to be shortened to approximately 5–15 min, which significantly reduces the risk of complications and improves procedural success rates [15,50]. Finally, an essential part of the process is the assessment of treatment outcomes after the intervention. Regular follow-up examinations and monitoring of healing processes enable early detection of potential complications. Technologies and methodologies employed in guided endodontics also ensure greater consistency and predictability of results [31]. Most studies focus on clinical evaluation, such as the absence of pain, other symptoms, and the stability of radiological findings like complete periapical healing. Radiographically, reductions in lesion size, return to normal periodontal ligament space, and the absence of new pathology are common indicators of successful healing. However, some studies lack detailed descriptions of clinical and radiological outcomes, highlighting the need for standardized reporting protocols. Importantly, while short-term assessments (e.g., 24 h, 1 week) can suggest positive results based on pain relief and absence of symptoms, only long-term follow-up (e.g., beyond six months) provides a reliable evaluation of the durability and stability of the treatment outcome. Currently, there are no standardized guidelines regarding the optimal duration and frequency of follow-up visits in guided endodontics, which limits the comparability of results across studies. More standardized measuring protocols and higher quality studies (such as randomized clinical trials) are needed to compare the results of GE. Standardization of disinfection protocols and obturation techniques in guided endodontics would enhance reproducibility and allow more reliable comparison between studies. Furthermore, detailed description of the complete clinical protocol is essential, including the stages following guided access preparation. After successful localization and negotiation of the newly created root canal, adequate chemical disinfection with irrigants such as sodium hypochlorite and EDTA, proper working length determination, mechanical preparation, and three-dimensional obturation must be clearly reported. In addition, analysis of the cost–benefit ratio of guided endodontics should be included. Although GE requires investment in CBCT imaging, digital planning software, CAD/CAM technology, and template fabrication, the reduction in chair time, decreased risk of iatrogenic errors (such as perforations or missed canals), and increased predictability of treatment may justify these costs, particularly in complex cases with pulp canal obliteration. A comprehensive evaluation comparing financial expenditure, clinical efficiency, and long-term outcomes would provide valuable insight into the practical applicability of this technique in daily practice.

This review has several strengths. First, it provides a comprehensive synthesis of available clinical in vivo studies on guided endodontics in cases of pulp canal obliteration. Second, it focuses not only on the technical aspects of the procedure but also on clinical workflow, CBCT parameters, instrument selection, and treatment outcomes, offering a structured overview of the current evidence. Additionally, the inclusion of detailed tables enhances transparency and facilitates comparison between studies. However, certain limitations must be acknowledged. The included studies were predominantly case reports and case series, with a lack of randomized controlled trials and long-term follow-up data. The heterogeneity in reported CBCT parameters, voxel sizes, planning software, bur dimensions, and outcome assessment criteria limited direct comparison between studies. Furthermore, inconsistent reporting of clinical and radiographic follow-up protocols reduced the ability to perform a standardized evaluation of long-term success. These limitations reflect the current stage of development of guided endodontics and highlight the need for higher-quality, standardized clinical studies.

## 5. Conclusions

In conclusion, although most studies report favorable outcomes, there is still a lack of precise, standardized timeframes and detailed guidelines for assessing the long-term efficacy of this method. Future efforts should focus on establishing unified protocols for technical parameters—such as CBCT settings, bur selection, rotational speed, software planning standards, and quality control intervals—to standardize procedures and improve comparability between clinical centers.

## Figures and Tables

**Table 1:** Digital and clinical workflow of guided endodontics.

1. LabolatoryPhase
CBCT & Digital Registration
Acquire CBCT scan (high resolution, small FOV, DICOM format)
Obtain digital arch registration (via intraoral scanner or impression & scanning) (STL format)
Virtual Planning
Overlay CBCT (DICOM) and arch STL in planning software
Localize visible root canal parts
Position virtual drill to reach root canal
Template Design
Design guide covering adjacent teeth surfaces
Adjust sleeve height and diameter for drill
Export STL of the guide
3D Printing
Process STL in slicer software
Print the guide
2. Clinical Phase
Guide Fit Check
Verify fit and stability
Preparation
Mark access point through guide
Remove enamel with diamond bur
Endodontic Procedure
Insert drill through guide hole
Move drill in depth with pumping movements
Clean drill regularly and irrigate root canal
Use hand file to check negotiation
Continue until the drill reaches sleeve dimension
Root Canal Treatment
Locate canal
Proceed with endodontic treatment

**Table 2:** Data extraction of the literature on guided endodontics in primary endodontic treatment of anterior teeth [27,28,29,30,31,32,33,34,35,36,37,38,39,40,41,42,43,44,45,46,47,48,49,50,51,52,53].

Author, Year	Teeth	Diagnosis	Problem	CBCT	FOV	Voxel Size	Impression	Scanner	Planning Software	Printer	Bur Type	Bur Specification	Bur Speed	Template Sleeve	Template Material	Time	Working Length	Planned Length of the Drill Path	Reached Canal Length
Nabavi et al. (2025) [52]	21	PN	PCO	Planmeca ProMAX 3D Classic; Planmeca Oy, Helsinki, Finland	No data	No data	No	Primescan scanner, Dentsply Sirona, Germany	Blue Sky Bio LLC, Glenview, Illinois, USA	Sonic 4K 3D Printer, Phrozen Technology, Hsinchu City, Taiwan	#1 Munce Discovery Bur (CJM Engineering, Santa Barbara, CA, USA)	No data	No data	No data	Resin	No data	No data	No data	No data
Kasar et al. (2025) [53]	21	No data	PCO	Planmeca ProMAX 3D Classic; Planmeca Oy, Helsinki, Finland	No data	No data	No	No data	3Shape Implant Studio (3Shape, Copenhagen, Denmark)	Ackuretta DENTIQ-120; Ackuretta Technologies, Taipei, Taiwan	Surgical bur (Mani Inc.; Tochigi, Japan	No data	No data	No data	No data	No data	22 mm	No data	No data
Kuczmaja et al. (2025) [27]	22	No data	PCO and root fracture	Carestream 9300 C, Carestream Dental2016, Atlanta, GA, USA	No data	No data	No	Carestream 360	Blue Sky Plan 4 software	Phrozen Sonic Mini 4K, Taipei, Taiwan	#1 Munce Discovery Bur (CJM Engineering, Santa Barbara, CA, USA)	No data	No data	5 mm L, 1.05 mm D	NextDent SG, Istanbul, Turkey	No data	No data	18 mm	No data
Fernández-Grisales et al. (2025) [34]	(a) 11 (b) 31 (c) 13 (d) 11	AAP	PCO	Planmeca ProMAX 3D Classic; Planmeca Oy, Helsinki, Finland	50 × 40 mm	0.075 mm	No	Medit i700; Medit, Seoul, Korea	Planmeca Romexis	No data	(a–c) Tivoly drill (d) DSP drilling system	(a) 0.75 mm D and 21 mm L (b) 0.75 mm D and 23 mm L (c) 0.9 mm D and 23 mm L (d) 0.8 mm D and 25.5 mm L	10,000 rpm	(a, b) Metal sleeve: 3.5 mm in external D, 0.75 mm in internal D, 5 mm L (c) Metal sleeve: 3.5 mm external D, 0.9 mm internal D, 5 mm L (d) No data	Surgical guide resin; Formlabs, Somerville, MA, USA	No data	No data	No data	Where the calclification-free canal could be identified (c) 14 mm, 19 mm—with template (d) 17 mm, 22 mm—with template
Abdulwahed et al. (2024) [35]	11	AAP	PCO	Orthophos S 3D, Dentsply Sirona, Bensheim, Germany	No data	No data	No	3Shape Trios, Copenhagen, Denmark	BlueskyPlan, Libertyville, IL	Asiga MAX, Asiga, Alexandria, NSW, Australia	Munce Discovery Bur (CJM Engineering, Santa Barbara, CA, USA)	No data	No data	No data	No data	No data	No data	9 mm (with template 16.01 mm)	No data
MahjourianQomi et al. (2024) [36]	(a) 31 (b) 41 (c) 42	(a) AAP (b) AAP (c) PN	PCO	Planmeca ProMAX 3D Classic; Planmeca Oy, Helsinki, Finland	No data	No data	No	3Shape Trios, Copenhagen, Denmark	Dental System v2017; 3Shape, Copenhagen, Denmark	Sonic 4K 3D Printer, Phrozen Technology, Hsinchu City, Taiwan	Munce Discovery Bur size #1 (CJM Engineering, Santa Barbara, CA, USA)	No data	No data	No data	Photopolymerized biocompatible polymer resin (PowerResins SG, Singapore)	No data	No data	(a, b) Between the middle and apical regions (c) Between the coronal and middle parts	No data
Fornara et al. (2024) [37]	33	SAP	PCO	Morita Veraview X800, J.Morita, Tokyo, Japan	6 × 7 cm	0.16 mm reconstruting to raw data with 0.08 mm	No	Aadva, IOS 100P, GC, Leuven, Belgium	Mimics—Materialise, Leuven, Belgium	Selective Laser Melting My Sint100 Sisma, Piovene Rocchette, Italy	210L16 205 008 Komet Dental Gear Brassier GmbH & Co. KG, Lemgo, Germany	0.8 mm D	No data	Sleveless	Grade 23 medical titanium	No data	No data	No data	No data
Valverde et al. (2024) [29]	31	SAP	PCO	GIANO HR, Newton, Imola, Italy	No data	No data	Adhesive pastes (President, Coltene, Altstätten, Switzerland)	AutoScan-DS-EX Pro, Shining 3D, Hangzhou, China	No data	No data	EG5 drill (Endoguide drill, SS White, Lakewood, CO, USA)	34 mm L 1.5 mm D	20 000 rpm	No data	No data	10 min	no data	In the middle third of the root	In the middle third of the root
Ambu et al. (2023) [38]	18 calcified single-rooted teeth	PN	PCO	Hyperion X 5 (MyRay, Cefla, Imola, Italy)	6 × 6 cm upper jaw 6 × 7 cm lower jaw	0.16 mm reconstruting to raw data with 0.08 mm	No	Aadva, IOS 100P, GC, Leuven, Belgium	RealGuide software (3diemme, Cantů, Italy)	SprintRay Pro 95 DLP Technology, SprintRay, Los Angeles, CA, USA	No data	22 mm L 0.75 mm D lower teeth 0.9 mm D upper teeth	10,000 rpm	5 mm L 3.5 mm external D 0.75 mm internal D lower teeth 0.9 mm D upper teeth	No data	No data	No data	Where the calcification-free canal could be detected	Teeth with optimal bur course: After reaching the WL with the bur, the canal was accessed and filled. Teeth with acceptable bur course: After reaching the bur length, the canal was further widened under the microscope using ultrasonic tips until patency was achieved
Lewis et al. (2023) [39]	22	PN with periapical cyst	PCO	iCAT; Imaging Sciences International, Hatfield, PA, USA	No data	No data	No	3Shape Trios, Copenhagen, Denmark	3Shape Implant Suite™	3Shape	ET bur	34 mm L 0.6 mm D	No data	No data	Resin	No data	No data	17 mm	15 mm
Zargar et al. (2023) [40]	21	AAP	PCO	NewTom Vgi, Verona, Italy	6 × 6 cm	0.1 mm	No	3Shape Trios, Copenhagen, Denmark	BlueskyPlan, Libertyville, IL	Dentafab, Istanbul, Turkey	Munce bur size 1 (Meisinger, Germany)	16 mm L 0.8 mm D	No data	Metal customized guiding sleeve 5 mm L 3 mm external D 1 mm internal D	Resin	No data	No data	6 mm above the apex	No data
Braga Diniz et al. (2022) [28]	(a) 12 (b) 11 (c) 12	(1) No data (2) AAP (3) Coronary fracture	PCO and previous iatrogenic deviation	iCAT; Imaging Sciences International, Hatfield, PA, USA	No data	0.12 mm	No	3Shape Trios, Copenhagen, Denmark	SimPlant Version 15.0 Pro; Materialise Dental, Leuven, Belgium	Objet Eden 260 V, Stratasys Ltd., Mineapolis, MN, USA	Neodent Drill for TempImplants, Ref.: 103179; JJGC Ind. E Comercio de Materiais Dentarios SA, Curitiba, Brazil	No data	1200 rpm	No data	MED 610	No data	No data	No data	No data
Nabavi et al. (2022) [41]	(a) 31 (b) 41 (c) 42	PN	PCO	Planmeca ProMAX 3D Classic; Planmeca Oy, Helsinki, Finland	No data	No data	No	3Shape Trios, Warren, NJ, USA	Dental System v2017; 3Shape, Copenhagen, Denmark	Sonic 4K 3D Printer; Phrozen Technology; Taiwan	Munce Discovery Bur size 1 (CJM Engineering, Santa Barbara, CA, USA)	0.7 mm D	No data	No data	No data	No data	No data	No data	No data
Loureiro et al. (2021) [30]	21	AAP	PCO	No data	No data	No data	Silicon Express XT, 3 M, Sumaré, Brazil	3Shape Trios, Copenhagen, Denmark	coDiagnostix (Dental Wings Inc., Montreal, QC, Canada)	Moonray DLP 3D-Printer (Sprintray, Los Angeles, CA, USA)	(1) Experimental diamond bur Helse Ultrasonic, Ribeirăo Preto, Brazil (2) Implant drill	(1) 1.5 mm D (2) 1.3 mm D	(2) 800 rpm	1.5 mm D guide tube (Neodent, Curitiba, Brazil)	Surgical guide resin (Sprintray, Los Angeles, CA, USA)	No data	No data	In the apical third	No data
Velmurugan et al. (2021) [42]	(a) 12 (b) 21 (c) 21	(a) SAP (b) SAP (c) SAP	PCO	Romexis software Planmeca, Finland	No data	0.075–0.15 mm	No	Shining 3D; Hangzhou, China	Geomagic Freeform plus; Haptic Technology 3D systems, Rock Hill, USA	Form2, Formlabs INC., Somerville, MA, USA	Gates-Glidden drill no. 2	0.8 mm D	No data	No data	Transparent resin	15 min	No data	No data	(a) In the middle third region (b) In the middle third region (c) No data
Llaquet Pujol et al. (2021) [43]	(a) 21 (b) 13 (c) 21 (d) 11 (e) 11 (f) 21 (g) 11	(a) SAP (b) CAA (c) AAA (d) SAP (e) AAA (f) AAP (g) AAP	PCO	Newtom5GXL; Newtom, Verona, Italy	No data	No data	No	3Shape Trios, Copenhagen, Denmark	BlueskyPlan, Libertyville, IL, USA	No data	Cylindrical diamond bur	21 mm L 1 mm D	10,000 rpm	No data	(a) PMMA (b) SLA (c) FDM (d) FDM (e) SLA (e) PMMA G PMMA	No data	No data	No data	No data
Freire et al. (2021) [44]	21	AAP	PCO	Prexion 3D Elite; Terarecon, SanMateo, USA	No data	0.1 mm	No	3Shape Trios, Holmens Kanal, Copenhagen, Denmark	ImplantViewer, Anne Solutions, Săo Paulo, Brazil	Form2, Formlabs INC., Somerville, MA, USA	Diamond bur Neodent, Ref. 103179; Curitiba, Brazil	20 mm L 1.3 mm D	10,000 rpm	No data	No data	No data	21.5 mm	No data	No data
Todd et al. (2021) [45]	22	PN, SAP	PCO	Dentsply Sirona	No data	No data	No	Cerec, Dentsply Sirona	Sicat Endo	No data	No data	24 mm L	No data	Metal (Sicat Endo)	No data	No data	21 mm	18 mm—from the incisal edge to the observable canal	18 mm
Ishak et al. (2020) [46]	(a) 31 (b) 41	PN	PCO	VGI evo, NewTom, Verona, Italy	24 × 19	0.2 mm	No	3Shape Trios, Copenhagen, Denmark	3shape, Copenhagen, Denmark	Asiga Max, Sydeny, Australia	FFDM, Pneumonat, Bourges, France	0.75 mm D	No data	5 mm L 1 mm external D 0.85 internal D	No data	No data	(a) 15.5 mm (b) 15 mm	(a) 9 mm (b) 4 mm	(a) 12.5 mm (b) middle third of the root
Silva et al. (2020) [47]	22	PN	PCO	No data	No data	No data	No	3Shape R700Scanner, Holmens Kanal, Copenhagen, Denmark	SimPlant Version 11; Materialise Dental, Leuven, Belgium	No data	Neodent Drill for TempImplants, refe. 103179, JJGC Ind and Dental Materials Trade SA, Curitiba, Brazil	20 mm L 1.3 mmD	10,000 rpm	No data	No data	No data	No data	Visible canal in the apical third of the root	No data
Hedge et al. (2020) [48]	11	AAP	PCO	PLANMECA	No data	No data	No	CS3500-KODAK, Carestream	BlueskyPlan, Libertyville, IL, USA	FormLabs2	Round bur (LN surgical round bur (Mani)	28 mm L 010–1mm D?	No data	Yes	Clear resin (FormLabs 2)	15 min	No data	No data	The junction of the middle and apical third
Buchgreitz et al. (2018) [49]	(a) 11, 21–17 (b) 12, 22–14 (c) 13, 23–5 (d) 31, 41–9 (e) 32, 42–4 (f) 33, 43-1	PCO with signs of SAP-44 PCO in need of a post–6	PCO	Orthophos XG 3D unit, Sirona Dental Systems, Bensheim, Germany	No data	0.5 mm	No	Cerec, Sirona Dental Systems	Galaxis/Galileos Implant, Sirona Dental Systems	CNC technology Sicat optiguide, Bonn, Germany	(1) Highspeed bur–enamel (2) Modified spiral bur (Busch, Engelskirchen, Germany)-dentine	(2) 1.2 mm D	(2) 250 rpm	Metal 1.2 mm internal D CNC technology Sicat optiguide	No data	No data	No data	The drill path was designed to reach the first visible part of the root canal	No data; better precision when length of the drill path is longer than pulp space obliteration
Torres et al. (2019) [31]	22	AAP	PCO,	VGI evo, NewTom, Verona, Italy	10 × 10 cm	0.2 mm	Alginate	Activity 885, SmartOptics, Bochum, Germany	Mimics Medical software 19.0 (Materialise, Leuven, Belgium) 3-matic Medical software 11.0 (Materialise)	Objet Connex 350 3D Printer (Stratasys, Eden Prairie, MN, USA)	Munce Discovery Bur size 1 (CJM Engineering, Santa Barbara, CA, USA)	34 mm L 0.8 mm D (head)	10,000 rpm	7 mm L 1 mm D	MED 610	No data	No data	No data	No data
Thorz et al. (2019) [32]	31	SAP	PCO	Orthophos SL, Dentsply Sirona, Bensheim, Germany	5 × 5 cm	0.08 mm	Alginate	No data	Sicat Endo	No data	Spiral carbide bur (Hager&meisinger, Neuss, Germany)	24 mm L 1.2 mm D	5000 rpm	Metal	No data	10 min to localize pulp space	No data	Apical third of the root	Shallower than virtually planned depth
Lara-Mendes et al. (2018) [50]	21	SAP	PCO	iCAT; Imaging Sciences International, Hatfield, PA	No data	0.12 mm	No	R700 Desktop Scanner (3Shape, Warren, NJ, USA)	SimPlant Version 11; Materialise Dental, Leuven, Belgium	Objet Eden 260 V, Stratsys Ltd., Mineapolis, MN, USA	(1) FG 1014 HL (KG Sorensen, Cotia, SP, Brazil)–enamel (2) Neodent Drill for TempImplants, Ref. 103179; JJGC Ind e Comercio de Materiais Dentarios SA, Curitiba, Brazil	(2) 20 mm total L, 12 mm working L 1.3 mm D	1200 rpm	Ref. 102110; JJGC Ind e Comercio de Materiais Dentarios SA 8 mm L 3 mm external D 1.4 mm internal D	FullCure 720	No data	No data	11.79 mm	No data
FonsecaTavares et al. (2018) [33]	(a) 11 (b) 11	(a) SAP (b) SAP	PCO	No data	No data	No data	Silicon	3Shape R700Scanner, Holmens Kanal, Copenhagen, Denmark	SimPlant Version 11; Materialise Dental, Leuven, Belgium	Objet Eden 260 V, Stratsys Ltd., Mineapolis, MN, USA	Neodent Drill for TempImplants Ref. 103179; JJGC Ind e Comercio de Materiais Dentarios SA, Curitiba, Brazil	20 mm L 1.3 mm D	10,000 rpm	Metal	FullCure 720	No data	No data	(a) Apical one third of the tooth	No data
Connert et al. (2018) [51]	(a) 31 (b) 41	(a) PN (b) SAP	PCO	Accuitomo 80; J. Morita Mfg. Corp., Irvine, CA, USA	No data	No data	No	iTero, AlignTechnology Inc., San Jose, CA, USA)	coDiagnostix (Dental Wings Inc., Montreal, SC, Canada)	Objet Eden 260 V, Stratasys Ltd., Mineapolis, MN, USA)	Gebr.Brasseler GmbH&Co. KG, Lemgo, Germany)	0.85 mm D	10,000 rpm	Steco–system–technik GmbH & Co. KG, Hamburg, Germany	MED 610	No data	No data	4 mm from the apex	No data
Krastl et al. (2016) [15]	11	SAP	PCO	Accuitomo 80; J. Morita Mfg. Corp., Irvine, CA, USA	50 × 50	0.08 mm	No	iTero, AlignTechnology Inc., San Jose, CA, USA)	coDiagnostix (Dental Wings Inc., Montreal, SC, Canada)	Objet Eden 260 V, Stratsys Ltd., Mineapolis, MN, USA	Straumann Drill for TempImplants, Ref.: 80381; Institut Straumann, Basel, Switzerland	37 mm total L 18.5 mm working L 1.5 mm D	10,000 rpm	Metal fabricated by CNC technology 6 mm L 2.8 mm extarnal D 1.5 mm internal D	MED 610	5 min–location of the root canal	24.4 mm	7.7 mm from the apex	9 mm from the apex, approximately 1 mm from the target point

Letter coding: PCO—pulp canal obliteration; SAP—symptomatic apical periodontits, AAP—asymptomatic apical periodontits, CAA—chronical apical abscess, AAA—acute apical abscess, PN—pulp necrosis. L—length, D—diameter; WL—working length. PMMA—polymethyl methacrylate, SLA—stereolithography resin, #—size1, FDM—fused deposition modeling.

**Table 3:** The clinical and radiological follow-up.

Authors, Year	Follow-Up: Time	Follow-Up: Pain	Follow-Up: Healing/Symptoms
Nabavi et al. (2025) [52]	18 months	Asymptomatic	The lesion had completely healed
Kasar et al. (2025) [53]	No data	No data	No data
Kuczmaja et al. (2025) [27]	3 months, 6 months, 1 year	No pain	No signs of discomfort, tenderness, or radiographic abnormalities
Fernández-Grisales et al. (2025) [34]	(a) 18 months, (b) 6 months (c) 18 months (d) 6 months	No data	(b) No data (a, c, d) Periapical healing achieved
Abdulwahed et al. (2024) [35]	1 year	No data	Complete healing
MahjourianQomi et al. (2024) [36]	6 months	Asymptomatic	A normal periodontal ligament space around the apex
Fornara et al. (2024) [37]	2 years	No pain	Complete healing
Valverde et al. (2024) [29]	3 years	absence of clinical symptoms	reduction in the size of the periapical reaction
Ambu et al. (2023) [38]	One week	No data	No data
Lewis et al. (2023) [39]	3 months	Asymptomatic	Tooth is functional as normal
Zargar et al. (2023) [40]	18 months	Asymptomatic	Apical lesion was healed
Braga Diniz et al. (2022) [28]	(a) 1 year (b) 1 year (c) 15 days	Asymptomatic	No data
Nabavi et al. (2022) [41]	6 months	Asymptomatic	Normal function
Loureiro et al. (2021) [30]	6 months	No symptoms	No signs of periapical pathology
Velmurugan et al. (2021) [42]	(a) 18 months (b) 1 year (c) 1 year	(a) Asymptomatic (b) Asymptomatic (c) Asymptomatic	No data
Llaquet Pujol et al. (2021) [43]	1 year	No pain	Periapical healing achieved
Freire et al. (2021) [44]	(a) 60 days (b) 2 years	(a) Discreet pain (b) Asymptomatic	(b) Asymptomatic healing
Todd et al. (2021) [45]	24 h	She was comfortable	No data
Ishak et al. (2020) [46]	No data	No data	No data
Silva et al. (2020) [47]	1 year	No data	Integrity of treatment
Hedge et al. (2020) [48]	No data	No data	No data
Buchgreitz et al. (2018) [49]	No data	No data	No data
Torres et al. (2019) [31]	6 months	The absence of symptoms	Completly healed periapical area
Thorz et al. (2019) [32]	No data	No data	No data
Lara-Mendes et al. (2018) [50]	1 year	Asymptomatic	Small alteration in the periodontal ligament space which may be a sign of scar tissue
FonsecaTavares et al. (2018) [33]	(a) 15 days (b) 30 days	Asymptomatic	No data
Connert et al. (2018) [51]	No data	No data	No data
Krastl et al. (2016) [15]	15 months	No pain	No signs of apical pathology

## Data Availability

No new data were created or analyzed in this study. Data sharing is not applicable to this article.
